# A Novel Fuzzy-Based Remote Sensing Image Segmentation Method

**DOI:** 10.3390/s23249641

**Published:** 2023-12-05

**Authors:** Barbara Cardone, Ferdinando Di Martino, Vittorio Miraglia

**Affiliations:** 1 Department of Architecture, University of Naples Federico II, Via Toledo 402, 80134 Naples, Italy; b.cardone@unina.it (B.C.); vittorio.miraglia@unina.it (V.M.); 2 Center for Interdepartmental Research “Alberto Calza Bini”, University of Naples Federico II, Via Toledo 402, 80134 Naples, Italy

**Keywords:** remote sensing, RSIS, fuzzy clustering, image segmentation, FGFCM, TCR

## Abstract

Image segmentation is a well-known image processing task that consists of partitioning an image into homogeneous areas. It is applied to remotely sensed imagery for many problems such as land use classification and landscape changes. Recently, several hybrid remote sensing image segmentation techniques have been proposed that include metaheuristic approaches in order to increase the segmentation accuracy; however, the critical point of these approaches is the high computational complexity, which affects time and memory consumption. In order to overcome this criticality, we propose a fuzzy-based image segmentation framework implemented in a GIS-based platform for remotely sensed images; furthermore, the proposed model allows us to evaluate the reliability of the segmentation. The Fast Generalized Fuzzy c-means algorithm is implemented to segment images in order to detect local spatial relations between pixels and the Triple Center Relation validity index is used to find the optimal number of clusters. The framework elaborates the composite index to be analyzed starting by multiband remotely sensed images. For each cluster, a segmented image is obtained in which the pixel value represents, transformed into gray levels, the graph belonging to the cluster. A final thematic map is built in which the pixels are classified based on the assignment to the cluster to which they belong with the highest membership degree. In addition, the reliability of the classification is estimated by associating each class with the average of the membership degrees of the pixels assigned to it. The method was tested in the study area consisting of the south-western districts of the city of Naples (Italy) for the segmentation of composite indices maps determined by multiband remote sensing images. The segmentation results are consistent with the segmentations of the study area by morphological and urban characteristics, carried out by domain experts. The high computational speed of the proposed image segmentation method allows it to be applied to massive high-resolution remote sensing images.

## 1. Introduction

Remotely sensed images are used increasingly in many problems related to the analysis and control of the territory, such as the analysis of climate risks on urban and natural fabrics and the control of the territory for the purposes of prevention from natural disasters or those generated by anthropic, soil protection, and environmental pollution control [1,2,3]. One of the critical points relating to the processing of remote sensed data is the fact that it is a massive amount of data and is continuously updated over time. This entails the need to use methods and techniques for processing remote sensed images which, on the one hand, optimize CPU times and memory allocation, and on the other provide accurate and reliable results. In particular, one of the most used image processing methods in remote sensing image analysis is image segmentation, which has the objective of partitioning the image into non-overlapping patterns having different characteristics. It allows you to detect and extract areas of the study area with specific characteristics (for example, soil types) [4].

Different Remote Sensing Image Segmentation (RSIS) methods have been proposed in the literature; among them, the most used are pixel-based RSIS algorithms, which use threshold and clustering analysis techniques to segment the image based on the pixel values. Threshold [5,6,7] is an RSIS technique in which optimal thresholds are obtained by dividing the image histogram into two or more parts; the Otsu method [8] is the most widely used RSIS threshold method. In clustering RSIS algorithms, a clustering method is applied to classify the pixels so that pixels assigned to the same cluster (segment) have characteristics that are as similar as possible and as dissimilar as possible to pixels assigned to other segments. K-means [9], Fuzzy C-means (FCM) [10], and their variants are the more used clustering algorithms applied in RSIS methods [11,12,13,14]. They are computationally very fast but are very sensitive to the presence of outliers and noises in the data and do not consider local spatial relations between nearest pixels; in addition, a validity index needs to be used to set the number of clusters.

Region-based RSIS methods are iterative methods in which adjacent regions of the image are merged to form larger regions. The main region-based RSIS methods are region grooving and region splitting and merging segmentation methods [15]. In RSIS region grooving algorithms, seed elements consisting of small regions of the image are initially selected; subsequently, each of these regions are enlarged by applying growth rules that merge adjacent pixels that have specific common characteristics. On the contrary, the region splitting and merging segmentation methods split heterogeneous regions into smaller regions; these methods do not require manual selection of seeds but are computationally more complex than region merging methods.

The best-known region grooving RSIS algorithm is JSEG [16]. JSEG is applied and uses the color and texture characteristics of the image to define the growth rules. JSEG is computationally fast but suffers from the problem of image over-segmentation. To overcome this problem, in [17] a hybrid JSEG algorithm based on wavelet transform, called WJSEG, was proposed; the results of tests performed on high-resolution SPOT 5 pan-sharpened multispectral images and IKONOS panchromatic images showed that JSEG provides more accurate segmentation results with respect to JSEG, reducing the over-segmentation problem. However, it is sensitive to noise and is computationally slow.

To improve the accuracy of the segmentation results, recently meta-heuristic RSIS methods were proposed. An ANN-based RSIS method using an enhanced boosted convolutional neural network was proposed by [18]. In [19], a lightweight deep learning noise robust image segmentation method is proposed to detect and measure dam crack widths. These methods are robust to noise and produce very accurate results but require numerous training data and the training process is computationally very expensive.

In [20], a hybrid thresholding image segmentation method based on an adaptive fractional-order particle swarm optimization algorithm was developed; the results of testing on samples of aerial images show that this method improves the segmentation performances of the Otsu thresholding RSIS algorithm. However, it is very slow in processing massive satellite images. An FCM-based RSIS algorithm in which features are extracted in the remote sensed image and used as samples by machine learning classifiers is proposed in [21]; this method considers some characteristics of the remoted sensed image as entropy, intensity, and edge features, but it neglects the local relations between pixels. Some authors developed variations of FCM for image segmentation which overcome some critical points, such as the neglect of the spatial relations between pixels and the lack of robustness with respect to the presence of noise and outliers. In [22,23], variations of FCM to increase the robustness are proposed. They improve the robustness to the noise of FCM; however, they do not consider the spatial relations between neighboring pixels.

An extension of FCM, called Fast Generalized Fuzzy *c*-means (FGFCM), was proposed in [24] to incorporate local spatial value information in the image. FGFCM was applied in [25] to segment images compressed by using the bidimensional Fuzzy Transform [26]. The authors show that this model provides a good trade-off between the segmentation accuracy and time and memory consumption. In [27], this image segmentation model was applied to segment medical massive bedsores images in order to monitor the status and evolution of bedsores in elderly people unable to access hospital facilities during the COVID-19 pandemic period. A variation of FGFCM is proposed in [28], in which the PSO algorithm is used to find the centers of the initial clusters to avoid FGFCM getting stuck at the local minimum. To improve the robustness with respect to noise in the image, a variation of FCM called Modified Robust Fuzzy C-Means (MRFCM) is tested in [29] to segment brain magnetic resonance (MR) images. MRFCM is more robust to various types of noise than other FCM-based image segmentation algorithms; however, it has a high computational complexity and is unsuitable for the processing of massive and multi-modal images. In [30], a variation of FGFCM called Generalized FCM aiming to be independent from the parameters used in FGFGM is proposed. The authors show that this algorithm provides results comparable with FGFGM; however, the CPU times required are too high to make it suitable for RSIS applications.

Therefore, recently proposed RSIS methods improve the accuracy and robustness to noise compared to canonical RSIS methods but are computationally expensive. In summary, recently proposed RSIS methods improve the accuracy and robustness of the FGFCM algorithm; in contrast, it is very fast but is less robust to noise. Furthermore, it depends on the selection of the number of clusters, which is fixed a priori. The main goal of this research is to test a new RSIS cluster-based method that provides a trade-off between the accuracy of the results and the computational speed and which is robust to the presence of noise in the images.

Moreover, since in many problems the segmentation process must be carried out on raster datasets that represent specific indices built starting from multiband source satellite images, the segmentation process must be executed for any type of raster dataset, which represents a particular index, regardless of the domain of values assumed by this index. In fact, generally, remotely sensed images are used in GIS-based applications in order to construct a composite index as a function of the image in a set of bands. For example, if we intend to analyze the spatial distribution of the Normalized Difference Vegetation Index (NDVI), which provides information on the health and density of vegetation covering a study area, using Landsat satellite images in the Red (R) and Near InfraRed (NIR) bands, it is possible to calculate the NDVI index using the formula NDVI = (NIR − R)/(NIR + R). The result is a raster dataset, i.e., a dataset in image format containing information belonging to any domain, in which the values of the cells range between the interval [−1, 1]. Of course, an image dataset is a type of raster dataset. Therefore, an RSIS method must be able to analyze any type of raster dataset, which represents a particular index.

For this purpose, a new GIS-based framework applied to satellite image segmentation based on FGFCM is proposed; a preprocessing phase is performed to create the raster dataset representing a composite index by using multiband remotely sensed images. This raster dataset is, then, transformed in an image dataset and the triple center relation (for short, TCR) clustering validity measure [31] is used to assess the optimal number of fuzzy clusters C. Subsequently, FGFCM is executed on the image representing the synthetic index to obtain C gray images where in the j*th* pixel of the i*th* image is stored the membership degree of the pixel to the i*th* cluster. Then, a final classified raster dataset is constructed in which the value of a pixel is given by the label of the cluster to which it belongs with the highest membership degree.

The proposed framework allows us to overcome the limitations of the RSIS methods proposed in the literature. In particular:

- It provides a method to segment any type of raster dataset representing a specific synthetic index so that its use is not restricted only to source remotely sensed images;
- The use of the FGFCM segmentation algorithm facilitates considering the relations between neighboring pixels, spatial constraints, and local spatial information in the image;
- The triple center relation validity index [31] determines the optimal number of clusters even in the presence of noisy images and cluster centers that are spatially close to each other. This feature is fundamental in cluster-based RSIS as remotely sensed images can be affected by various types of noise.

In summary, the proposed RSIS framework, unlike the RSIS models proposed in the recent literature, maintains the high computational speed of the FGFCM algorithm; furthermore, it is more robust than FGFCM with respect to the presence of noise in the image, providing more accurate results. Finally, it can be applied to any type of raster dataset constructed from the source multiband satellite image.

The rest of this paper is organized as follows: in Section 2 the FGFCM clustering image segmentation method and the TCR validity index are briefly described. Section 3 introduces the proposed framework and describes in detail its functional components. Section 4 presents and discusses the results of our tests performed on remotely sensed images. The conclusions are presented in Section 5.

## 2. Preliminaries

In this section, the RSIS FGFGM algorithm is synthetized and the TCR validity index used in our framework in a preprocessing phase to set the optimal number of fuzzy clusters is briefly described.

### 2.1. The FGFCM Image Segmentation Algorithm

The FGFCM algorithm is proposed in [24] to incorporate local spatial and grey level information together.

Let **X** = {x_1_,...,x_N_} ⊂ R^n^ be a dataset of N elements where each element is a point in the space R^n^ of the n features. If the dataset is a gray image having N pixel, n = 1 and the element x_j_ is given by the gray value of the j*th* pixel.

Let **V** = {v_1_,…,v_C_} ⊂ R^n^ the C cluster centers to be detected.

To consider local information, in [24] the following transformation to the jth element is performed:

(1) $$\xi_{j}=\frac{\sum_{k\in N_{w}}S_{\mathrm{jk}}x_{k}}{\sum_{k\in N_{w}}S_{\mathrm{jk}}}$$

where N_w_ is a window around the jth pixel and the weight S_jk_ is given by

(2) $$S_{\mathrm{jk}}=\left\{\begin{array}{l} S_{s\_\mathrm{jk}}\cdot S_{g\_\mathrm{jk}}\;\mathrm{if}\;k\neq j\\ 0\;\mathrm{if}\;k=j \end{array}\right.$$

in which the term S_s_jk_ measures the influence of the kth pixel in the set of the neighbors to the jth pixel and the term S_g_jk_ measures the grey similarity.

The term S_s_jk_ is given by

(3) $$S_{s\_\mathrm{jk}}=\exp\left(\frac{-\max\left(\left|p_{j}-p_{k}\right|,\left|q_{j}-q_{k}\right|\right)}{\lambda_{s}}\right)$$

where (p_j,_ q_j_) and (p_k,_ q_k_) are the coordinate, respectively, of the jth and the kth pixel and λ_s_ sets the spread of the exponential function.

The term S_g_jk_ is given by

(4) $$S_{g\_\mathrm{jk}}=\exp\left(\frac{-\|x_{j}-x_{k}\|{}^{2}}{\lambda_{g}\cdot \sigma_{g\_j}^{2}}\right)$$

where λ_g_ sets the spread of the function S_g_jk_. The parameter σ_g_j_ is a function of the density of the local region surrounding the jth pixel; the higher this density, the higher its value. It is defined as

(5) $$\sigma_{g\_j}=\sqrt{\frac{\sum_{k\in N_{w}}\|x_{j}-x_{k}\|{}^{2}}{N}}$$

The objective function to minimize is

(6) $$J\left(X,U,V\right)=\overset{C}{\underset{i=1}{\sum }}\overset{q}{\underset{r=1}{\sum }}\gamma_{r}u_{\mathrm{ir}}^{m}{\left(\xi_{r}-v_{i}\right)}^{2}$$

where q < N is the number of distinct grey level values in the transformed image, γ_r_ is the number of pixels in the transformed image having grey level r, and ξ_r_ is the value of the lth grey level in the transformed image.

Applying the Lagrange multiplier method to find the minimum of (6), the solutions for U and V are obtained:

(7) $$u_{\mathrm{ir}}=\frac{{\left(\xi_{r}-v_{i}\right)}^{-\frac{2}{m-1}}}{\sum_{k=1}^{C}{\left(\xi_{r}-v_{k}\right)}^{-\frac{2}{m-1}}}$$

and

(8) $$v_{i}=\frac{\sum_{r=1}^{q}\gamma_{r}u_{\mathrm{ir}}^{m}\xi_{r}}{\sum_{r=1}^{q}\gamma_{r}u_{\mathrm{ir}}^{m}}$$

where u_ir_ is the membership degree of the pixels having value ξ*_r_* to the i*th* cluster and v_i_ is the center of the i*th* cluster.

In output, FGFCM provides C images with N pixels, where the i-th image represents, transformed in the interval [0, 255], the degree of belonging of the pixel to the ith cluster.

Below is shown in pseudocode the FGFCM algorithm (Algorithm 1).
**Algorithm 1:** FGFCM   *Input:*   Original image with N pixels I   Number of clusters C   Fuzzifier m   End iteration threshold ε   *Output:* The C segmented images1. *Initialize* randomly the center of the clusters c_i_ i = 1,…,C2. **For** j = 1,…,N3. *Transform* the value of the j*th* pixel by (1)4. q:= number of distinct grey level values in the transformed image5. **Repeat**6.     **For** i = 1,…,C7.    **For** r = 1,…,q8.     *Compute* u_ir_ by (7)9.    **Next** r10.     *Compute* v_i_ by (8)11.    **Next** **i**12. **Until** $\left|U^{\left(t\right)}-U^{\left(t-1\right)}\right|>\varepsilon$ $\left|U^{\left(t\right)}-U^{\left(t-1\right)}\right|>\varepsilon$13. **For** i = 1,…,C14.  Create the i*th* segmented image15. **Next** i16. **Return** the C segmented images

### 2.2. The TCR Validity Index

The TCR index is a fuzzy clustering validity measure related to the well-known Dunn index [32] used to detect compact well-separated clusters. The TCR is applied to assess the compactness of clusters and the separability among clusters.

Let **X** = {x_1_,...,x_N_} ⊂ R^n^ be a dataset of N elements where each element is a point in the space R^n^ of the n features.

Let **V** = {v_1_,…,v_C_} ⊂ R^n^ the C cluster centers.

The mean and the variance of the cluster centers are defined as

(9) $$\hat{v}=\frac{1}{C}\overset{C}{\underset{i=1}{\sum }}v_{i}$$

and

(10) $$\sigma_{v}^{2}=\frac{1}{C-1}\overset{C}{\underset{i=1}{\sum }}\|v_{i}-{\hat{v}\|}^{2}$$

The compactness of the cluster is measured by the following index:

(11) $$\mathrm{Com}\left(C\right)=\overset{C}{\underset{i=1}{\sum }}\frac{\sum_{j=1}^{N}u_{\mathrm{ij}}^{m}\|x_{j}-v_{i}{\|}^{2}}{\sum_{j=1}^{N}\underset{i=1,..,C}{\max}u_{j}^{m}}$$

The separability among clusters is measured by the following indices:

(12) $$\mathrm{Sep}\left(C\right)=S_{1}\left(C\right)\cdot S_{2}\left(C\right)\cdot S_{3}\left(C\right)$$

where

(13) $$S_{1}\left(C\right)=N\cdot \sigma_{v}^{2}$$

(14) $$S_{2}\left(C\right)=\frac{1}{C}\overset{C}{\underset{i=1}{\sum }}\overset{C}{\underset{\begin{array}{c} k=1\\ k\neq i \end{array}}{\sum }}\|v_{i}-v_{k}\|{}^{2}$$

(15) $$S_{3}\left(C\right)=\underset{i=1,\ldots ,C}{\min}\overset{C}{\underset{\begin{array}{c} k=1\\ k\neq i \end{array}}{\sum }}\|v_{i}-v_{k}\|{}^{2}$$

The three indices measure, respectively, the sample variance, the mean distance among cluster centers, and the minimum distance among cluster centers. Their combination obtains accurate measurements of intra-cluster separability, even in cases where the cluster centers are closely distributed. The lower the value of SEP(C), the higher the intra-cluster separability.

The final TCR index is given by the ratio between the compactness and the separability indices:

(16) $$\mathrm{TCR}\left(C\right)=\frac{\mathrm{Com}\left(C\right)}{\mathrm{Sep}\left(C\right)}$$

The optimal number of clusters is selected by minimizing the TCR index. In Algorithm 2 the algorithm using TCR to find the optimal number of clustering is shown in pseudocode, where any FCM-based algorithm can be used.

The results of tests performed in [19] show that TCR give better performances with respect to other fuzzy clustering validity indices in the presence of noised datasets.
**Algorithm 2:** TCRValidityIndex   *Input:*    Dataset with N elements D        Fuzzifier m        End iteration threshold ε   *Output:* Optimal number of clusters1. Set C_MAX_     //maximum value for the number of clusters2. C_OPT_:= 1      //initialization of the best number of clusters3. TCROLD:= 0  //initialization of the TCR4. **For** c = 1,…,C_MAX_5.   Execute FCM-based algorithm (D, c, m, ε)6.   Compute Com(c) by (11)7.   Compute Sep(c) by (12)8.   TCR = Com/Sep   //TCR index obtained for c clusters9.   **If** c = 1 **Then**10.        TCR_OLD_ = TCR11.    **Else**
12.     **If** TCR < TCR_OLD_ **Then**13.     C_OPT_:= c14.     TCR_OLD_ = TCR15.     **End if**16.    **End if**17. **Next** c18. **Return** C_OPT_

## 3. The Proposed Framework

**Figure 1:**
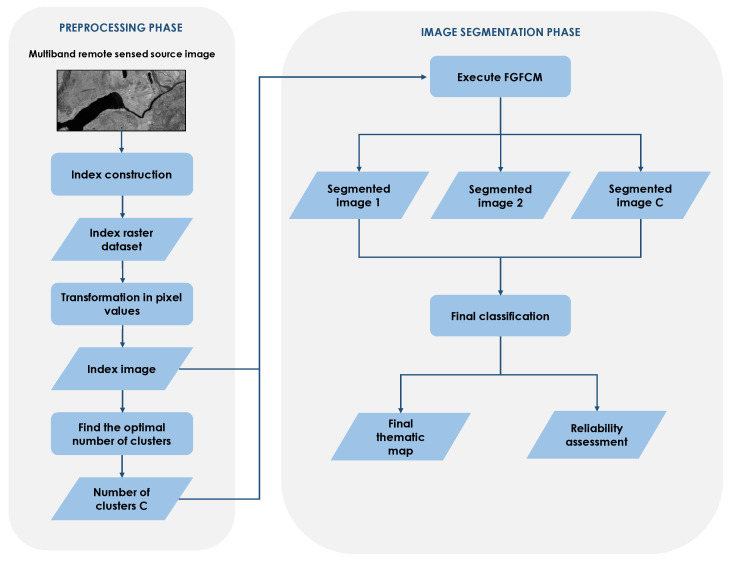
Schema of the proposed framework.

The proposed RSIS framework includes:

- A preprocessing phase in which, starting from the multiband remotely sensed image source, the raster dataset of a composite index is constructed and the TCR validity measure to find the optimal number of clusters is used;
- The image segmentation phase in which the FGFCM algorithm is executed to the index image and the final classified image is created.
- Figure 1 schematizes the architecture of the framework.

The source dataset is given by a set of remotely sensed images acquired in one or more bands. The *index construction* component is the GIS-based process in which raster functions and map algebra operators are used to compute the composite index raster dataset.

The *transformation in pixel values* component transforms the index domain in a digital image domain. For example, the NDVI raster dataset is transformed in an image dataset converting the range [−1, 1] in the range [0, 255]. The result of the process is an image in which the pixel values are made up of the transformed values of the index to be analyzed (*Index image*).

The framework is highly flexible so as to allow segmentation of the source image into a band as well. In this case, the Index Image consists of the source image in the specified band.

The final functional component (*Find the optimal number of clusters*) aims to determine the optimal number of fuzzy clusters using the TCR validity index. This component executes iteratively FGFCM, setting a different number of clusters each time and measuring the corresponding TCR value. The number of clusters C chosen is the one that minimizes the TCR index.

**Figure 2:**
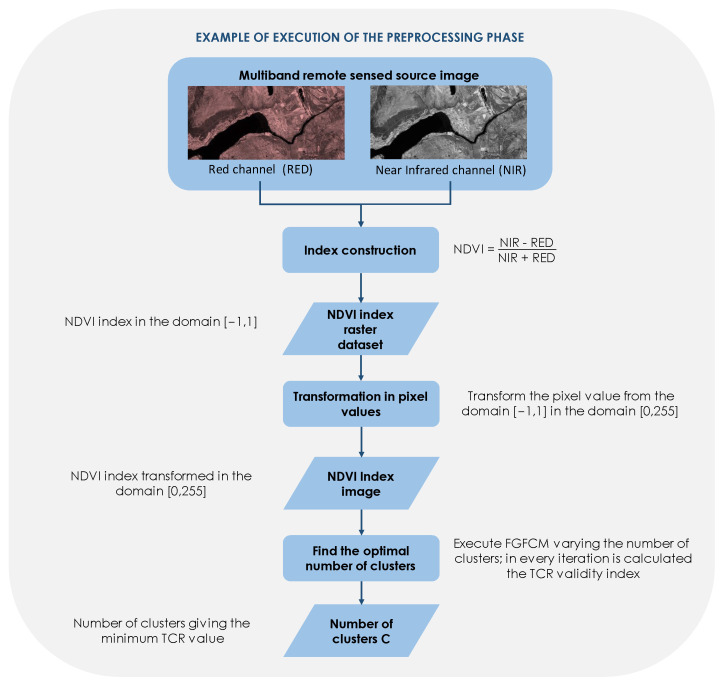
Example of execution of the preprocessing phase.

An example of execution of the preprocessing phase in which the raster dataset of the NDVI index is created is schematized in Figure 2.

In the image segmentation phase FGFCM is executed on the index image, setting the number of fuzzy clusters to C. Outputs of the component *Execute FGFCM* are the set of C segmented images where the value of a pixel in the i*th* segmented image are converted in the digital image domain from the membership degree of the pixel to the i*th* cluster.

The *Final classification* component assigns to each pixel the label of the cluster to which it belongs with the highest degree of membership. The component provides a raster dataset in which the pixel values are given by the classes they belong to. A thematic map is appropriately constructed creating a one-to-one association between a cluster and a thematic class and assigning a semantic label to the thematic class. (*Final thematic map*). In addition, for each thematic class, the reliability of the assignment of pixels to the corresponding cluster is evaluated as the average of the membership degrees to the cluster of all the pixels assigned to it; the final assessed reliabilities are assigned to all the thematic classes and stored (*Reliability assessment*). The reliability measures for each cluster allows us to evaluate the reliability of the assignment of image pixels to the cluster; in fact, it is calculated as the average value of the membership degrees to the cluster of the pixels assigned to it. The higher this value, the greater the certainty that the pixels assigned to the cluster belong to it; therefore, the greater the accuracy of the detected segments.

Formally, if N_i_ is the number of pixels assigned to the i*th* cluster, the reliability of the assignment of these pixels to this cluster is given by

(17) $${\mathrm{Rel}}_{i}=\frac{1}{N_{i}}\overset{N_{i}}{\underset{j=1}{\sum }}u_{\mathrm{ij}}$$

Below is shown in pseudocode our RSIS method (Algorithm 3). FGFCM is the FCM-based algorithm used executing the TCRValidityIndex algorithm.
**Algorithm 3:** The proposed RSIS method* Input:  Original multiband image with N pixels* * Output: Final classification thematic map and reliability assessment*1.   *Set* m, ε2.*----------------   Preprocessing phase -------------------------------------*3.   *Construct* the composite index raster dataset CI4.   *Transform* the composite index raster dataset in an image dataset II5.   C:= TCRValidityIndex(II, m, ε)6.*----------------   Image segmentation phase -----------------------------*7.   Execute FGFCM(II, C, m, ε)8.   **For** j = 1,…,N9.           u_MAX_:= u_1j_10.         RCj:= l_1_ //label of the first cluster11.         **For** i = 2,…,C12.           **If** u_ij >_ u_MAX_ **Then**13.             u_MAX_:= u_ij_14.             RCj:= lb_i_ //label of the i*th* cluster15.           **End if**16.         **Next** i17.   **Next** j18.   **For** i = 1,…,C19.       Rel_i_:= 020.       Num_i_:= 021.       **For** j = 1,…,N22.          **If** RCj = lb_i_ **Then**23.            Rel_i_ = Rel_i_ + RCj24.            Num_i_ = Num_i_ + 125.          **End if**26.        **Next j**27.            Rel_i_ = Rel_i_/Num_i_28.   **Next** i29.   **Return** thematic map RC[N] and cluster assignment reliability Rel[C]

The framework was implemented in the ESRI ArcGIS desktop suite by using the Python ArcPy library.

In next section, we show the results of a set of tests of our framework applied on a study area given by the southwestern districts of Naples, Italy.

## 4. Test Results

The framework was tested on a study area given by the three districts of the southwestern area of the metropolitan city of Naples, Italy: Bagnoli, Fuorigrotta, and Posillipo.

**Figure 3:**
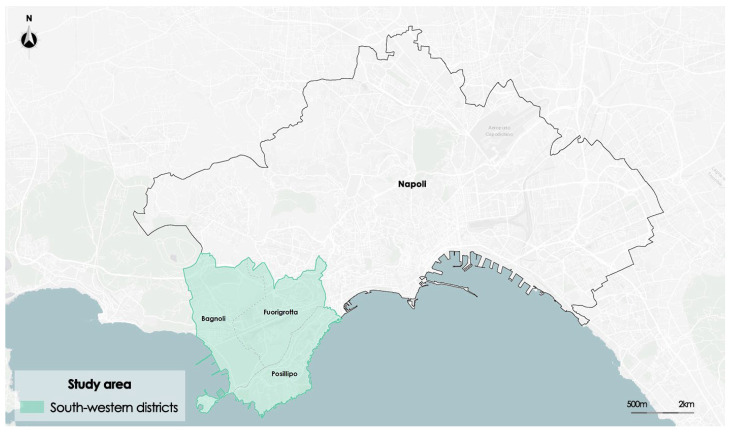
Framing of the study area: southwestern districts of Naples, Italy.

Figure 3 shows the study area that includes the three districts. The area has been identified in order to test the accuracy of the image segmentation process of raster data representing composite indexes extracted by satellite images.

### 4.1. Morphological Analysis

To improve our understanding of the data from satellite images, we have been provided a morphological description of the whole study area, thanks to an experienced planner.

Posillipo has a very mountainous landscape; the Coroglio ridge, which runs the entire length of the district, is the morphological feature that indicates the district’s division from the other two districts. All of Fuorigrotta is straight, with the exception of the eastern border region. The Agnano basin is a largely level volcanic area that is part of the Bagnoli district in the Campi Flegrei volcanic area. The southern area of the district is completely flat; almost all of the area is covered by an old industrial plant, now decommissioned for about 30 years, belonging to the old steel Italsider company.

**Figure 4:**
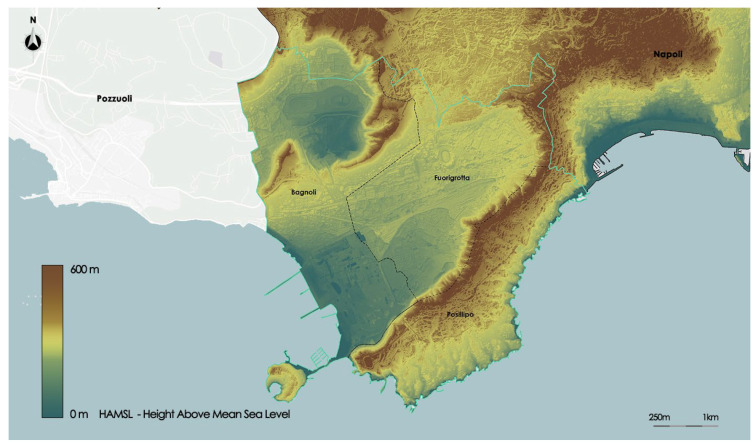
Digital Terrain Model of the study area.

To better understand the morphological constitution of the territory, in Figure 4 is shown the study area map of the Digital Terrain Model (DTM); a topographical model of the Earth’s surface that contains data, in a digital format, of the elevation of the bare ground devoid of any natural or anthropic element present on the surface. For the study area, the DTM domain has an interval between 0 and 600 m that measures the surface height above sea level.

The results obtained by running the proposed RSIS method on raster datasets of composite indices processed starting from satellite images are shown below. For brevity’s sake, we show the results obtained for three composite indices: Albedo, NDVI, and Sky View Factor.

The Albedo index identifies the fraction of light on a horizontal surface that is reflected in all directions; it constitutes the reflective power. It is aimed at identifying the reflection characteristic of the solar radiation affecting the materials on the ground. It takes values in the range [0, 1]. The maximum albedo is 1 when all the incident radiation is reflected; this occurs in the case of perfectly white soils. The minimum albedo is 0 when no fraction of the radiation is reflected; this value is obtained in the presence of perfectly black soils. The Albedo index was calculated as the weighted average of the ratios between the visible and near infrared (0.315–2.8 µm) incident and reflected energy, using the visible and infrared emission and absorption spectral bands obtained with the RapidEye satellite, with resolution of 7 × 9 m.

**Figure 5:**
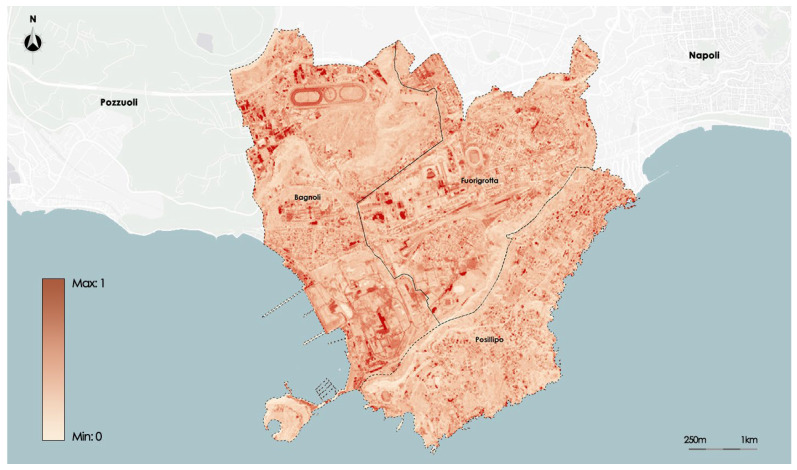
Map of Albedo satellite data.

Figure 5 shows the distribution of the Albedo on the study area.

The NDVI—Normalized Difference Vegetation Index—measures how vigorous the vegetation is. Its purpose is to document the presence of vegetation on the surface of the earth as well as its development over time. The ratio between the difference and a sum of the reflected radiation in the near infrared (NIR), in which the light is reflected by the leaves, and in the red (RED), in which the chlorophyll absorbs light, is used to compute the NDVI. The domain values are in the range of −1 and 1. When vegetation is present, values between 0.2 and 1 are assumed. The range of values between −1 and 0 can be attributed to uncultivated environments like streams and urban areas. The data are processed by the satellite Sentinel2 with a resolution of 7 × 7 m.

**Figure 6:**
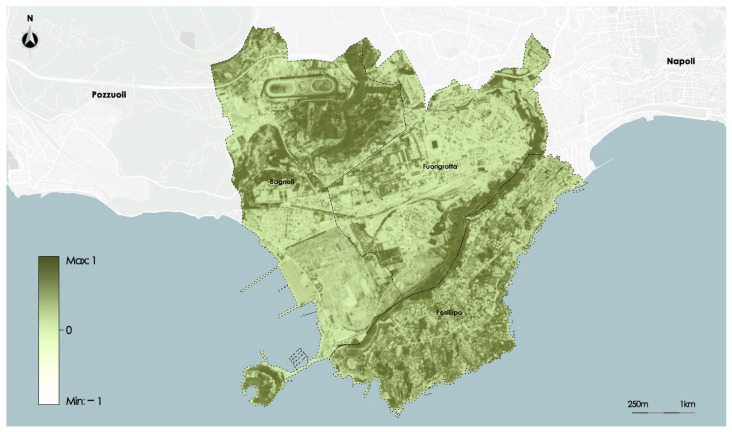
Map of NDVI satellite data.

Figure 6 shows the distribution of the NDVI on the study area.

The Sky View Factor (SVF) index indicates the fraction of sky visible from a point on the surface. The index shall be calculated taking into account any obstacle that prevents the full visibility of the sky. The domain is between 0 and 1. With the approximation of the values to 0, there is a smaller portion of the visible sky and an increasingly complete obstruction of visibility; with the application of the values to 1, it will increase the portion of the sky detectable until a complete visibility of 360°. This shows that the higher the SVF value, the greater the heat loss in the atmosphere. The values were processed by the satellite Landsat 8 with a resolution of 1.7 × 1.7 m.

**Figure 7:**
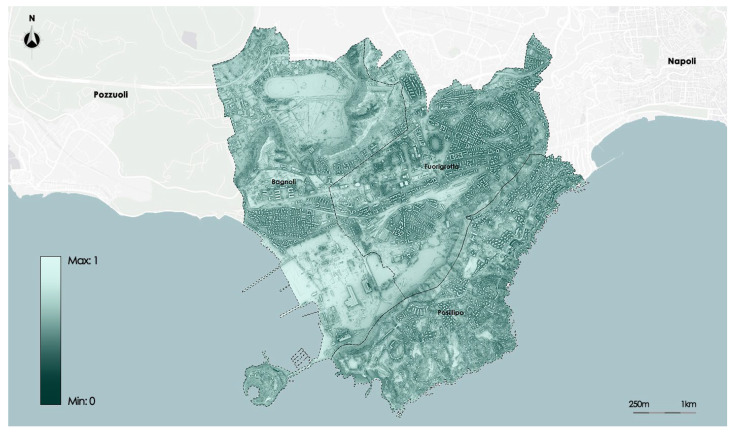
Map of Sky View Factor satellite data.

Figure 7 shows the distribution of the SVF on the study area.

Following the segmentation process, thematic maps for each index were created: Albedo, NDVI, and SVF.

**Figure 8:**
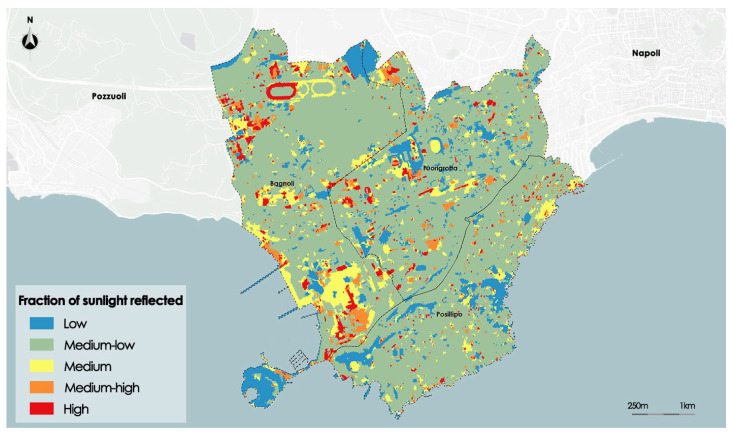
Map of Albedo after the segmentation process.

The optimal number of clusters determined in the preprocessing phase for the Albedo index is five. After executing the segmentation process, a thematic map of Albedo given by five thematic classes called, respectively, Low, Medium-Low, Medium, Medium-high, and High is created. Figure 8 shows the thematic map of the Albedo.

The segmentation algorithm was able to clearly distinguish areas with different values, managing to faithfully perimeter the areas as identified by the input raster. The inability to discern minute differences in values between several locations is the only drawback. According to the morphological analysis, it is clear that the areas with a lower value of Albedo are distributed mainly to the south along the ridge of Posillipo and north along the side of Mount Spina that delimits the basin of Agnano (locality of the district of Bagnoli).

The highest values are mainly concentrated within the complex of the Mostra d’Oltremare in the district of Fuorigrotta and in the disused industrial areas of the former Italsider and in the automotive sector of via Pisciarelli, respectively, to the south and north-east of Bagnoli.

The reliabilities assessed for each class are given in Table 1.

The average reliability is higher than 0.65 for all thematic classes, except the *Medium-low* thematic class, whose average reliability is equal to 0.58; furthermore, this thematic class presents the highest standard deviation of reliability. This is presumably due to the fact that this class includes large areas with different shapes and types of soil.

**Figure 9:**
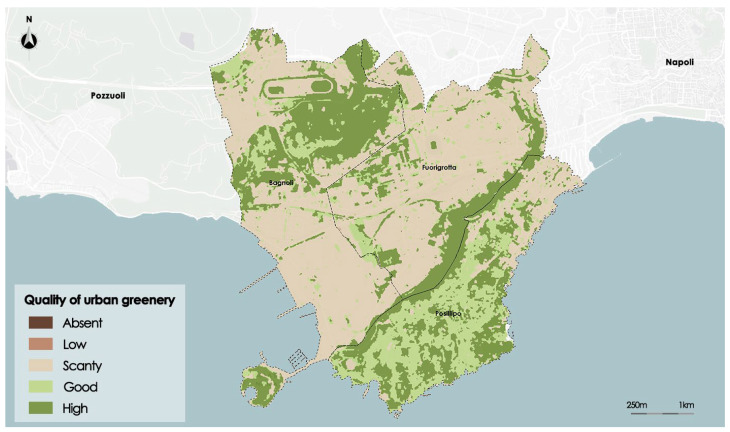
Map of NDVI after the segmentation process.

Now the results obtained for the NDVI index are shown. The optimal number of clusters determined in the preprocessing phase for the NDVI index is five. After executing, then in the segmentation process a thematic map of NDVI given by five thematic classes called, respectively: *Absent*, *Low*, *Scanty*, *Good,* and *High* is created. Figure 9 shows the thematic map of NDVI.

The technique for segmentation conformed to the same input raster’s boundary while accurately identifying areas with varying NDVI values. As per the planner’s expectations, the areas with the highest value correspond to the long ridge that splits the district of Posillipo from that of Fuorigrotta and to the basin of Agnano close to the border between the district of Bagnoli and Fuorigrotta. Both surfaces are mainly covered by wooded areas. Due to its high level of urbanization, the majority of the land is categorized as *Scanty*; both built or and natural surfaces belong into this class. However, the disused industrial area in Bagnoli is an example of how badly vegetated this class is.

The reliabilities assessed for each class are given in Table 2.

The average reliability is higher than 0.70 for all thematic classes except the *Scanty* thematic class, whose average reliability is equal to 0.55; furthermore, this thematic class presents the highest standard deviation of reliability (0.13). In fact, very large zones of the study area belong to this class, with a sparse presence of living vegetation, both in the built fabric and in impervious open spaces and in uncultivated or abandoned areas.

**Figure 10:**
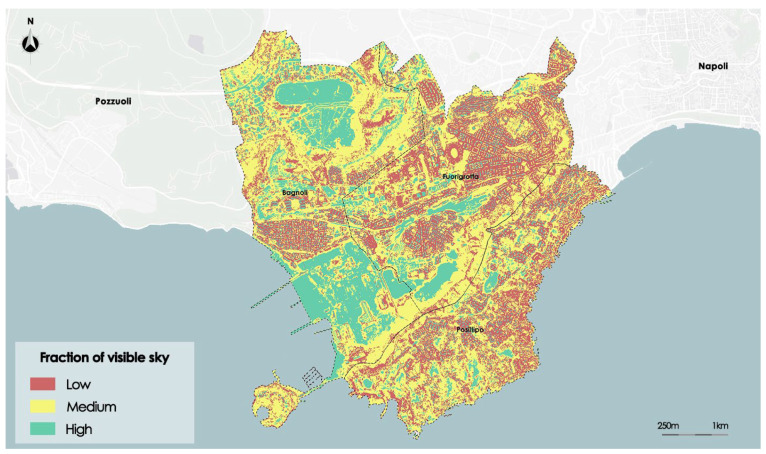
Map of Sky View Factor after the segmentation process.

Below the results obtained for the SVF index are shown. The optimal number of clusters determined in the preprocessing phase for the SVF index is three. After executing, then in the segmentation process a thematic map of Albedo given by three thematic classes called, respectively, *Low*, *Medium,* and *High* is created. Figure 10 shows the thematic map of the Sky View Factor.

Even more accurately than in the prior instances, the segmentation algorithm has captured the perimeter in this instance as well. The input file’s higher resolution than the other two raster images could be the cause of this. In line with the morphological analysis, the areas with higher values of SVF are those with a flat character, such as the disused industrial area in Bagnoli to the south and the flat inside the basin of Agnano to the north. Both areas have a high degree of visible sky fraction. As expected, the areas with the lowest level of visibility are those with a high density of built surfaces due to the dense mesh of buildings that hinders the fraction of sky visible from the road.

Table 3 shows the reliabilities assessed for three SVF thematic classes.

The mean reliability and the standard deviation of the three thematic classes is very similar; in particular, the mean reliability is higher than 0.7 for all thematic classes. This result highlights that areas with *Low*, *Medium,* and *High* sky view factors are very distinct from each other.

In order to analyze the performance of the proposed method, it was compared with the well-known Otsu thresholding segmentation method, analyzing a specific region of the study area selected by the domain experts. The comparison was performed by measuring the Hamming Distance [33] between the segmentation results obtained executing the Otsu thresholding algorithm and the proposed method. The Hamming distance between two binary segmentations R and S in a region evaluates the similarity between the two segmentations in that region. It is defined as

(18) $$\mathrm{HD}\left(R,S\right)=1-\frac{\left|R_{B}\cap S_{F}\right|+\left|R_{F}\cap S_{B}\right|}{\left|R\right|}$$

where $\left|R\right|$ is the number of pixels in the region, $\left|R_{B}\cap S_{F}\right|$ is the number of pixels of the region classified in the background in the segmentation R and in the foreground in the segmentation S, and $\left|R_{F}\cap S_{B}\right|$ is the number of pixels of the region classified in the background in the segmentation S and in the foreground in the segmentation R.

HD ranges between 0 and 1. The more HD approaches 1, the more similar the two segmentations are in the region of the analyzed image.

The two methods are executed to a selected region in the images of the three composite indexes of Albedo, NDVI, and Sky View Factor. To obtain the background and the foreground areas using our FGFCM-based segmentation method, the thematic classes in the resultant segmented image were aggregated to form only the two thematic classes called, respectively, *Foreground* and *Background*.

**Figure 11:**
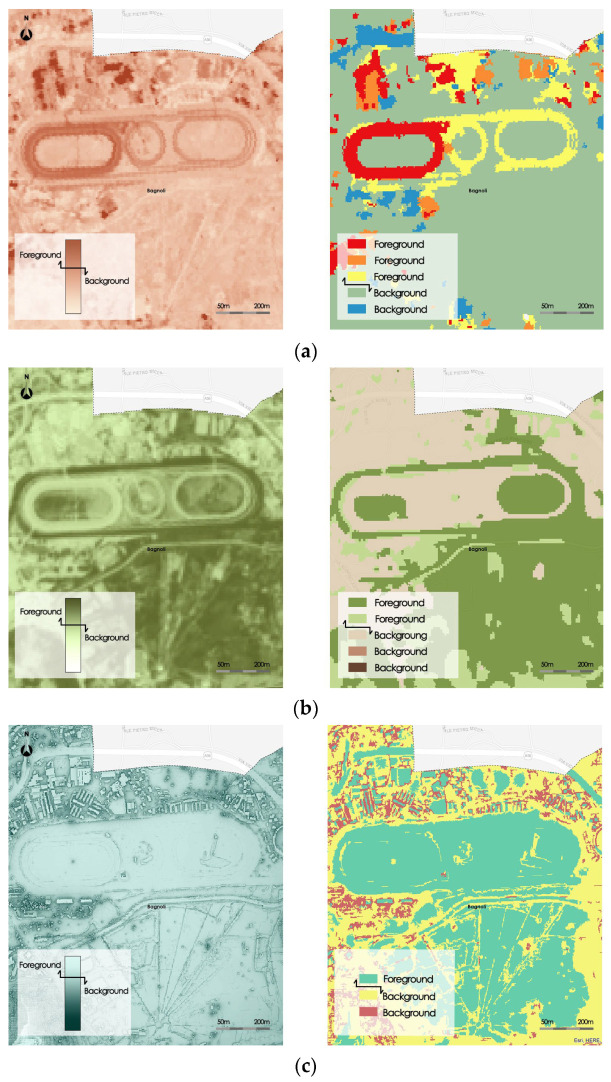
Segmented regions compared for the three synthetic indices: (**a**) Albedo, (**b**) NDVI, (**c**) Sky View factors.

Figure 11 show the segmentations obtained for the three synthetic indices analyzed: Albedo, NDVI, and Sky View Factor.

Table 4 shows the results of the comparison. The table shows the Hamming distance similarity measure and the execution times of the two methods necessary for the segmentation of regions in the three raster datasets.

The HD measure is higher than 0.9 in all three cases. Furthermore, the execution times obtained with the proposed method are in all cases lower than those obtained by running the Otsu algorithm.

### 4.2. Discussion of the Results

The results of the classification agree with assessments provided by topic-matter specialists who assessed how closely the areas described in the thematic map conformed to their morphological and urban features. This implies that the method proposed by us can be used to improve the analysis of urban systems thanks to its short computational time.

In fact, our algorithm can guarantee excellent results even with high-resolution satellite images without having to wait as long as other models do. From a classification point of view, our model allows the determination of the optimal number of clusters thanks to the use of the TCR validity index. This is guaranteed even in high-noise conditions.

By analyzing the low standard deviation values of each class found in each of the satellite rasters analyzed, it is possible to demonstrate that our model has a good degree of reliability in the determination of thematic classes and a low level of uncertainty.

Furthermore, the results of comparisons with the Otsu thresholding algorithm show that the proposed RSIS method provides good accuracy and better execution times.

## 5. Conclusions

A new RSIS method based on the Fast Generalized Fuzzy C-means algorithm is proposed. In a preprocessing phase, a raster dataset representing the distribution of the composite index on the study area is obtained by processing remotely sensed image datasets and the TCR validity index is used to determine the optimal number of clusters. Then, FGFCM is executed to obtain the segmented images; the segmentation result is given by a thematic map of the composite index in which each thematic class is related to a specific fuzzy cluster. A pixel is assigned to the thematic class corresponding to the cluster to which it belongs with the greatest membership degree. Finally, the mean reliability of every thematic class is assessed as the average membership degrees of the pixels belonging to the class.

Our framework was tested on a set of remotely sensed images to construct a segmented thematic map of composite indices in the study area given by the southwestern districts of Naples, Italy. The final thematic maps of the analyzed composite indices are in line with the assessments made by domain experts who evaluated the adherence of the areas classified in the thematic map with their morphological and urban characteristics.

The use of the FGFCM algorithm, which has a high computational speed, allows the proposed method to be applied also to high-resolution remotely sensed images; furthermore, the use of the TCR validity index can determine the optimal number of clusters even in the presence of noisy images. A further benefit is the assessment of the reliability of the final thematic classes, which allows the effectiveness of the classification to be assessed.

Our model, thanks to its ability to process remote sensing images at high resolutions in short computational times, can be a useful supporting tool for urban morphological analysis for the assessment of physical vulnerability compared to multi-risks caused by extreme events such as heatwaves or pluvial flooding.

In the future, we intend to carry out further comparative tests on different types of territories and urban settlements in order to determine the accuracy and efficiency of the proposed method as the type of study area and the resolution and the quality of the source remotely sensed images vary.

## Figures and Tables

**Table 1:** Reliabilities of the classes of Albedo.

Class	Mean Reliability	Standard Deviation
Low	0.74	0.11
Medium-low	0.58	0.07
Medium	0.77	0.08
Medium-high	0.75	0.07
High	0.67	0.08

**Table 2:** Reliabilities of the classes of NDVI.

Class	Mean Reliability	Standard Deviation
Absent	0.78	0.04
Low	0.71	0.08
Scanty	0.55	0.13
Good	0.68	0.09
High	0.72	0.08

**Table 3:** Reliabilities of the classes of SVF.

Class	Mean Reliability	Standard Deviation
Low	0.73	0.06
Medium	0.71	0.06
High	0.70	0.07

**Table 4:** Hamming distance and CPU time of the Otsu thresholding and the proposed method.

Synthetic Index	HD	Otsu CPU Time (s)	Our Method CPU Time (s)
Albedo	0.91	2.01	1.38
NDVI	0.93	2.14	1.42
Sky View Factor	0.95	1.97	1.40

## Data Availability

Data are contained within the article.
